# Supplementary material for: Anti-high Mobility Group Box 1 Antibody Ameliorates Albuminuria in MRL/lpr Lupus-Prone Mice
Source: Mol Ther Methods Clin Dev. 2017 May 25;6:31–9. doi: 10.1016/j.omtm.2017.05.006 (PMC5472134; doi:10.1016/j.omtm.2017.05.006)
Supplement: Document S1. Figure S1 [file mmc1.pdf]

OMTM, Volume *6*

Supplemental Information

Anti-high Mobility Group Box 1 Antibody Ameliorates Albuminuria in MRL/*lpr* Lupus-Prone Mice

Haruki Watanabe, Katsue S. Watanabe, Keyue Liu, Sumie Hiramatsu, Sonia Zeggar, Eri Katsuyama, Noriko Tatebe, Akiya Akahoshi, Fumiaki Takenaka, Takahisa Hanada, Masaru Akehi, Takanori Sasaki, Ken-ei Sada, Eiji Matsuura, Masahiro Nishibori, and Jun Wada

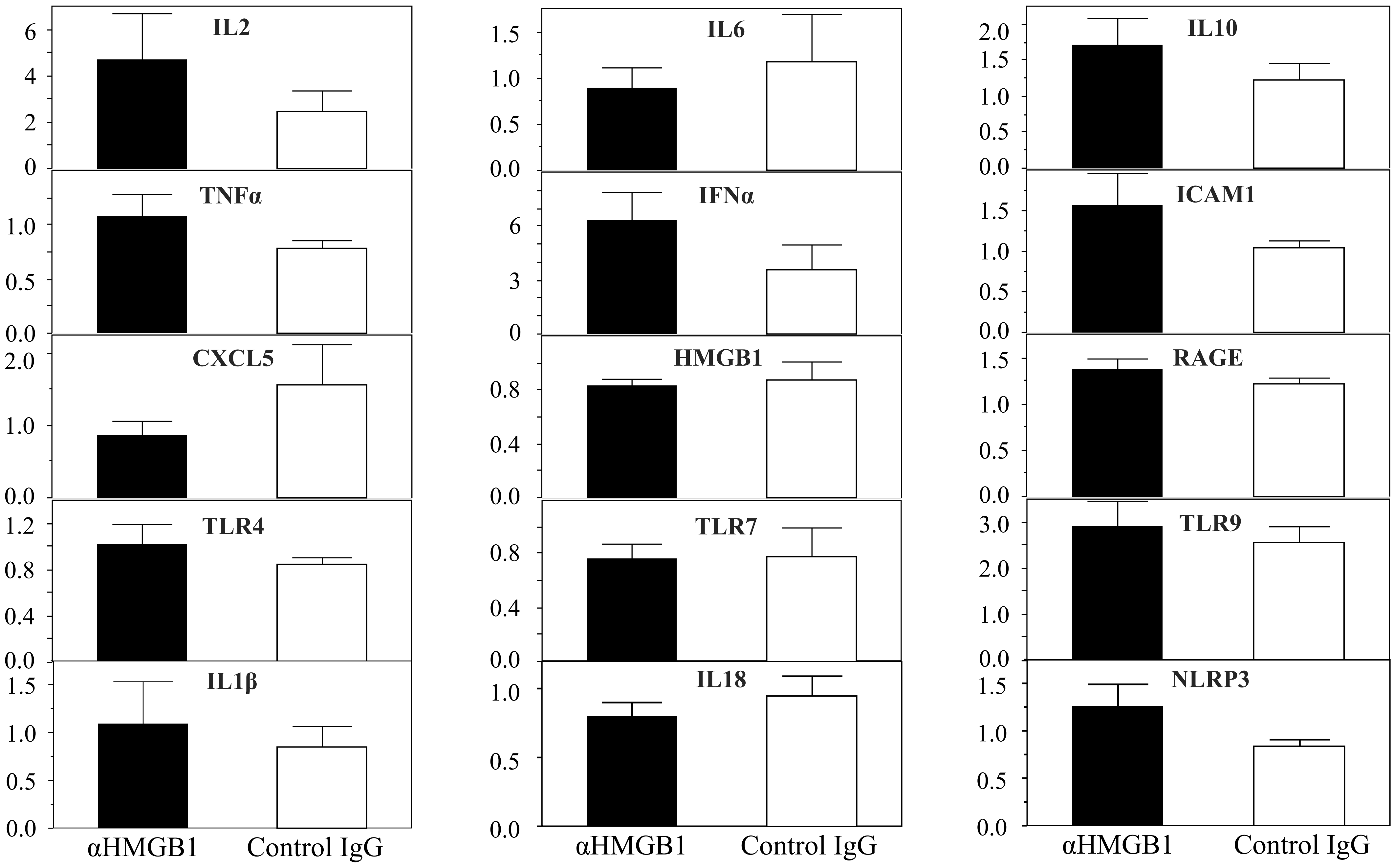

IL2
TNFα
CXCL5
TLR4
IL1β
IL6
IFNα
HMGB1
TLR7
IL18
IL10
ICAM1
RAGE
TLR9
NLRP3
αHMGB1
Control IgG
αHMGB1
Control IgG
αHMGB1
Control IgG

Supplemental Figure 1. Renal mRNA expression

Relative mRNA expression was evaluated by real-time RCR (n = 9 [IL2: n = 6, IL10, TNF α , ICAM, TLR4: n = 10]). Relative mRNA abundance was standardized using GAPDH and 18S mRNA as the invariant control. IL, interleukin; TNF, tumor necrosis factor; IFN, interferon; ICAM1, intercellular adhesion molecule 1; CXCL5, C-X-C motif chemokine 5; HMGB1, high mobility group box 1; RAGE, receptor for advanced glycation endproducts; TLR, toll-like receptor; NLRP3, NACHT, LRR and PYD domains-containing protein 3.
