# Supplementary material for: Anti-high Mobility Group Box 1 Antibody Ameliorates Albuminuria in MRL/lpr Lupus-Prone Mice
Source: Mol Ther Methods Clin Dev. 2017 May 25;6:31–9. doi: 10.1016/j.omtm.2017.05.006 (PMC5472134; doi:10.1016/j.omtm.2017.05.006)
Supplement: Document S2. Article plus Supplemental Information [file mmc3.pdf]

Molecular Therapy
Methods & Clinical Development
Original Article

Anti-high Mobility Group Box 1 Antibody Ameliorates Albuminuria in MRL/*lpr* Lupus-Prone Mice

Haruki Watanabe,[1] Katsue S. Watanabe,[1] Keyue Liu,[2] Sumie Hiramatsu,[1] Sonia Zeggar,[1] Eri Katsuyama,[1] Noriko Tatebe,[1] Akiya Akahoshi,[3] Fumiaki Takenaka,[3] Takahisa Hanada,[3] Masaru Akehi,[3] Takanori Sasaki,[3] Ken-ei Sada,[1] Eiji Matsuura,[3] Masahiro Nishibori,[2] and Jun Wada[1]

[1]Department of Nephrology, Rheumatology, Endocrinology and Metabolism, Okayama University Graduate School of Medicine, Dentistry and Pharmaceutical Sciences, Okayama 700-8558, Japan; [2]Department of Pharmacology, Okayama University Graduate School of Medicine, Dentistry, and Pharmaceutical Sciences, Okayama 700-8558, Japan; [3]Collaborative Research Center for OMIC, Okayama University Graduate School of Medicine, Dentistry, and Pharmaceutical Sciences, Okayama 700-8558, Japan

We evaluated the efficacy of a neutralizing anti-high mobility group box 1 (HMGB1) monoclonal antibody in MRL/*lpr* lupus-prone mice. The anti-HMGB1 monoclonal antibody (5 mg/kg weight) or class-matched control immunoglobulin G2a (IgG2a) was administered intravenously twice a week for 4–15 weeks. Urine albumin was monitored, and histological evaluation of the kidneys was conducted at 16 weeks. Lymphadenopathies were evaluated by 1-(2′-deoxy-2′-[^{18}F]fluoro-β-D-arabinofuranosyl)cytosine ([^{18}F]FAC) positron emission tomography/computed tomography (PET/CT) at 12 weeks. Following 4-week treatment, [^{18}F]FAC-PET/CT showed similar accumulation in cervical and axillary lymph nodes at 12 weeks of age. However, anti-HMGB1 monoclonal antibody sufficiently inhibited the increase in albuminuria compared to an isotype control following 15-week treatment. Complement deposition was also improved; however, there were no significant differences in IgG deposition and renal pathological scores between the two groups. Anti-double-stranded DNA (dsDNA) antibody titers and cytokine and chemokine levels were also unaltered. Although there were no significant differences in glomerular macrophage infiltration, neutrophil infiltration was significantly decreased by the anti-HMGB1 monoclonal antibody. Antagonizing HMGB1 treatment suppressed HMGB1 translocation from nuclei in the kidney and suppressed neutrophil extracellular traps. The anti-HMGB1 monoclonal antibody demonstrated therapeutic potential against albuminuria in lupus nephritis by inhibiting neutrophil recruitment and neutrophil extracellular traps.

INTRODUCTION

Lupus nephritis (LN) is a refractory complication of systemic lupus erythematosus (SLE), which causes end-stage renal disease, resulting in lower survival rates and quality of life.[1] The pathogenesis of LN has yet to be fully elucidated; however, it is assumed that innate and adaptive immunity abnormalities are associated with chronic systemic inflammation.[2] Although novel biologics targeting B or T cells have emerged, randomized controlled trials have failed to show their effectiveness against LN.[1]

Impaired clearance of apoptotic cells in lupus patients has been reported;[3] the consequently released DNA and nuclear proteins activate the innate immunity. High mobility group box 1 (HMGB1) is a ubiquitous non-histone nuclear protein that regulates transcription, repair, and recombination by exerting effects on chromosomal architecture.[4,5] In addition, HMGB1 can actively or passively translocate from the nucleus to the extracellular space under inflammation conditions; extracellular HMGB1 exerts pleiotropic effects as an alarmin-mediating inflammation through toll-like receptors (TLRs), receptor for advanced glycation end products (RAGE), and cytosolic DNA/RNA sensors or by promoting apoptosis and autophagy.[4–7] The redox status of HMGB1 distinguishes its cytokine-inducing and chemokine activity.[8,9] HMGB1 has also been shown to promote type 1 interferon (IFN) production through the TLR9 and RAGE pathways in response to DNA.[10] Type 1 IFN is known as a crucial cytokine in lupus;[11] therefore, these data suggest that HMGB1 is involved in lupus pathogenesis. Indeed, elevated concentrations of HMGB1 are observed in the sera, urine, kidney tissues, and skin lesions of patients with lupus.[12–14]

The relationship of HMGB1 with other autoimmune diseases, such as rheumatoid arthritis (RA), has also been suggested. HMGB1 is more strongly expressed in the synovial fluid of RA patients than in that of osteoarthritis patients, inducing the release of proinflammatory cytokines from synovial fluid macrophages.[15] In addition, HMGB1 boosts proinflammatory cytokine and matrix metalloproteinase production in synovial fibroblasts from RA patients.[16]

Received 20 March 2017; accepted 21 May 2017;
http://dx.doi.org/10.1016/j.omtm.2017.05.006.

Correspondence: Katsue S. Watanabe, MD, PhD, Department of Nephrology, Rheumatology, Endocrinology and Metabolism, Okayama University Graduate School of Medicine, Dentistry and Pharmaceutical Sciences, 2-5-1 Shikata-cho, Kita-ku, Okayama 700-8558, Japan.
E-mail: kwatanabe@md.okayama-u.ac.jp

This is an open access article under the CC BY license (http://creativecommons.org/licenses/by/4.0/).

Table 1. Organ Weights

	αHMGB1	Control
Total body weight	36.8 ± 0.8	36.1 ± 0.8
Right kidney	0.20 ± 0.009	0.20 ± 0.009
Left kidney	0.19 ± 0.008	0.20 ± 0.008
Spleen	0.44 ± 0.05	0.37 ± 0.05
Thymus	0.07 ± 0.005	0.08 ± 0.005
Peritoneal lymph nodes	1.9 ± 0.15	1.7 ± 0.15
Cervical lymph nodes	0.83 ± 0.11	0.74 ± 0.11
Axillary lymph nodes	0.65 ± 0.07	0.59 ± 0.07
Total lymph nodes[a]	3.37 ± 0.3	3.06 ± 0.3

Data presented are the mean ± SE of 19 mice per group. Weight values are in grams.
[a]Total lymph nodes, the combination of peritoneal, cervical, and axillary lymph nodes.

Thus, HMGB1 plays a pivotal role in the pathogenesis of autoimmune diseases and may constitute a potential therapeutic target. Interestingly, HMGB1 inhibition by a specific antibody has been shown to alleviate lupus-like disease in BXSB mice.[17] In contrast, a different study has recently reported that treatment with an anti-HMGB1 monoclonal antibody (mAb) does not affect lupus activities in MRL/*lpr* mice.[18] To elucidate this discrepancy, we examined the efficacy of anti-HMGB1 mAb to determine whether it ameliorates lupus activities, including nephritis and serological abnormalities, in MRL/*lpr* lupus-prone mice. Our mAb recognizes the C-terminal sequence of the HMGB1 molecule and can neutralize the intercellular adhesion molecule 1 (ICAM1)-inducing activity of HMGB1 in vitro;[19] moreover, therapeutic effects against brain stroke, atherosclerosis, and viral infections have also been reported.[19–21]

RESULTS

Organ Weights and Lymphoid Tissue Functions

There were no significant differences in organ weight at 16 weeks (Table 1) following 12 weeks of treatment. Next, we evaluated the lymphoid organs using 1-(2′-deoxy-2′-[^{18}F]fluoro-β-D-arabinofuranosyl)cytosine ([^{18}F]FAC) positron emission tomography/computed tomography (PET/CT) at an early stage. FAC accumulates in T cells; therefore, this imaging analysis enabled us to evaluate not only the sizes of organs, but also their functions.[22] [^{18}F]FAC PET/CT imaging analysis at 12 weeks was similar in the cervical and axillary lymph nodes of the two groups (Figure 1A). In addition, the amount of incorporated probe was also similar in the cervical lymph nodes and spleen (Figure 1B). These results indicate that lymphoid tissue weight and functions, especially of those of the T cells, were unaltered.

Serological Abnormalities and the Effects of the Anti-HMGB1 mAb

As shown in Figure 2A, anti-HMGB1 mAb treatment did not result in any significant reduction in anti-double-stranded DNA (dsDNA) antibody titers at 16 weeks. Although the plasma levels of a number of cytokines, such as interleukin-1β (IL-1β), IFN-γ, and tumor necrosis factor alpha (TNF-α), tended to decrease, there were no significant differences (Figures 2B and 2C). In addition, chemokine levels were also unaltered (Figure 2B). Moreover, antagonizing HMGB1 treatment did not result in a significant reduction in plasma HMGB1 levels (Figure 2D).

Urinary Albumin Excretion and Renal Pathological Evaluation

The anti-HMGB1 mAb sufficiently inhibited the increase in albuminuria compared to the increase observed for an isotype control at 16 weeks ($p = 0.008$, Figure 3A). Consistent with albuminuria, glomerular complement deposition also improved (Figure 3B). However, there were no significant differences in immunoglobulin G (IgG) deposition and renal pathological scores (activity index) between the two groups (Figures 3C and 3D).

Glomerular Cell Infiltration

To investigate the mechanism underlying the favorable renal effects of anti-HMGB1 mAb without serological improvement, we focused on glomerular inflammatory cells. No significant differences in the glomerular infiltration of F4/80-positive macrophages were apparent, whereas the infiltration of Ly-6G-positive neutrophils was significantly suppressed ($p = 0.034$, Figures 3E and 3F).

Renal mRNA Expression

We hypothesized that certain types of in situ cytokines or chemokines may drive neutrophil infiltration; however, renal mRNA expression was not suppressed by anti-HMGB1 mAb treatment (Figure S1). The expression of HMGB1 and its receptors was also similar (Figure S1). Although HMGB1 is an important factor in innate immunity, the expression of inflammasome-related genes was also unaltered (Figure S1).

Effect on HMGB1 Translocation and Neutrophil Extracellular Traps

Next, we evaluated whether the anti-HMGB1 mAb suppresses HMGB1 translocation from the nucleus in the kidney to determine the mechanism by which inflammation is prevented. Immunofluorescence staining revealed that treatment with this antibody suppressed translocation compared to the control antibody ($p = 0.035$, Figure 4A). However, the HMGB1 translocation appeared to be extensive in both treated mice groups compared to non-treated control mice (Figure 4A).

Neutrophil extracellular traps (NETs) were originally identified as a system for degrading virulence factors and killing bacteria via neutrophils;[23] however, recent studies have suggested their involvement in autoimmunity, including lupus and vasculitis.[24,25] Therefore, we also evaluated the change of NETs in the kidney. Immunofluorescence staining revealed that the anti-HMGB1 mAb successfully suppressed the manifestation of NETs in the glomerulus ($p = 0.022$, Figure 4B).

DISCUSSION

Our data demonstrate that the anti-HMGB1 mAb ameliorates albuminuria through inhibition of neutrophil infiltration, HMGB1

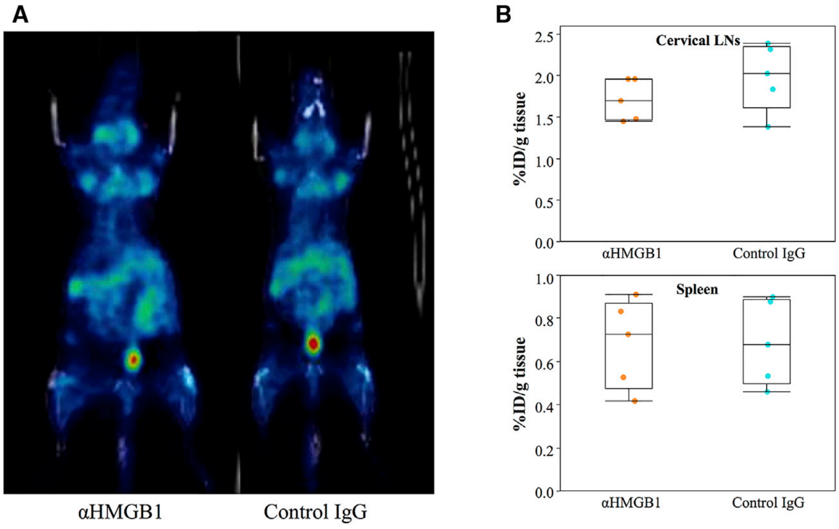

Figure 1. Accumulation of FAC in Lymphoid Tissues

(A) PET/CT images. (B) Incorporated probe in lymphoid tissues. Units signify % injected dose/gram tissue (%ID/g). Five mice per group were examined. LN, lymph node.

translocation, and NETs compared to control IgG treatment, without serological improvement.

With respect to the mechanism of action of the anti-HMGB mAb, we initially examined its inhibitory effect against neutrophil infiltration in glomeruli. Neutrophils are the first line of defense against bacterial and fungal infections;[26] they are rapidly recruited from the blood stream into sites of inflammation. Neutrophils induce responses in inflamed tissues to destroy the microorganisms through chemokine and cytokine release, phagocytosis and reactive oxygen species formation, and degranulation and NETs during infection.[27] In addition to these primary effectors against microbial pathogens, neutrophils are also central mediators of sterile inflammatory injury, suggesting they constitute a key player in autoimmune diseases.[28,29] Neutrophils are indeed activated in SLE, and increased numbers of abnormal neutrophils or low-density granulocytes in the circulation have been observed.[11,30] Cell death by necrosis or abnormal apoptosis releases multiple endogenous pro-inflammatory damage-associated molecular patterns, including proteins, nucleic acids, and lipid mediators, and then leads to neutrophil recruitment.[28,31] Anti-HMGB1 mAb could interact with these dangerous molecules by inhibiting HMGB1 translocation and interrupting neutrophil recruitment for local inflammation. Furthermore, Huebener et al.[32] recently reported that HMGB1 triggers infiltration of neutrophils toward acetaminophen-induced liver necrosis through its receptor RAGE, which may further confirm our findings.

The second mechanism explaining the efficacy of the anti-HMGB mAb is its inhibitory effects against NET formation in glomeruli. Increased formation of NETs due to impairment in their degradation has been reported in lupus.[33] NETs promote type I IFN production and therefore may be involved in pathogenesis.[34] Peptidylarginine deiminase (PAD) 4 plays a fundamental role in NET formation, including hypercitrullination of histone H3.[35] Inhibition of PAD4 disrupts NET formation and shows therapeutic potential for lupus activities in various mouse models.[36,37] Thus, NETs may constitute a promising target for mitigating SLE. An interaction between NETs and HMGB1 has also been reported. Tadie et al.[38] demonstrated that HMGB1 enhanced the formation of NETs through TLR4 and detected extensive citrullinated H3 in HMGB1-treated neutrophils, which were significantly suppressed by neutralizing antibodies against HMGB1. Another study revealed that HMGB1 and histones released by injured hepatocytes during ischemia/reperfusion injury stimulated NET formation through TLR4 and TLR9-MyD88 signaling pathways.[39] MRL/*lpr* mice also exhibit a vasculitis phenotype, and an in vitro study has revealed the linkage between HMGB1 and NETs in anti-neutrophil cytoplasmic antibody (ANCA)-associated vasculitis (AAV).[40] HMGB1 can potentiate ANCA-induced NET formation through TLR2, TLR4, and RAGE and may be involved in the pathogenesis of AAV.[41] On the basis of these previous findings, anti-HMGB1 mAb may suppress NETs by inhibiting neutrophil infiltration as well as by direct inhibition through HMGB1 receptors.

The effects of antagonizing HMGB1 treatment for arthritis have been reported as being generally beneficial.[42,43] Moreover, Zhang et al.[17] demonstrated the effectiveness of the anti-HMGB1 antibody for lupus in BXSB mice; they peritoneally injected 30 μg of anti-HMGB1 antibody every week from 16 to 26 weeks. Although the antibody dose seems considerably low, they showed attenuated proteinuria, glomerulonephritis, anti-dsDNA, and serum cytokine levels. However, a recent study has reported that the anti-HMGB1 antibody did not affect lupus activities in MRL/*lpr* mice.[18] Although this previous study used the same mouse model, the treatment protocol was different from our protocol, i.e., an antibody dose of 100 μg/mouse/week was used in contrast to our dose of approximately 150–400 μg/mouse/week, with peritoneal administration and a treatment period of 7–17 weeks. Therefore, the total administered dosage was smaller and the maximum blood concentration might be lower than that of our protocol. Moreover, the antibody used in the previous study recognizes the A-box,[7] which is different from the antibody used in our study raised against the c-tail epitope. The A-box domain antagonizes inflammation; therefore, A-box inhibition might exacerbate the disease.[5] Collectively, their treatment might not be sufficient to result in any advantages. In contrast, BXSB mice exhibit TLR pathway abnormalities; therefore, the former study could demonstrate favorable results despite the lower treatment dose than the other protocol. Our treatment protocol was determined based on previous studies.[19–21] Moreover, we first tried subcutaneous injection using the same dosage (5 mg/kg) as a preliminary

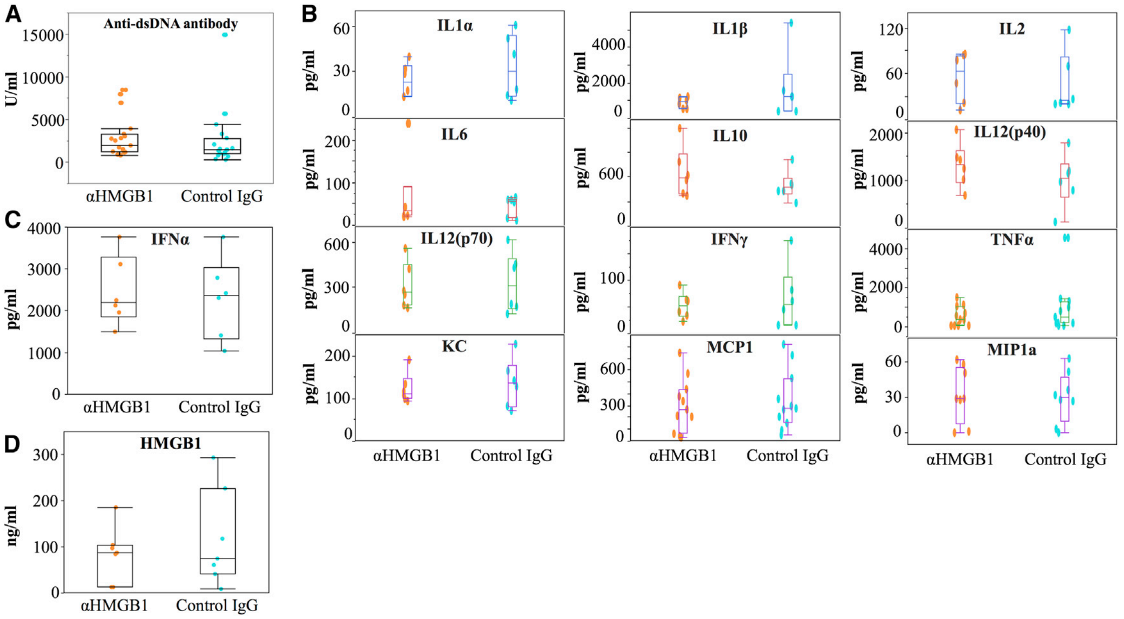

Figure 2. Effects of Anti-HMGB1 Monoclonal Antibody on Autoantibody Production and Inflammation in MRL/*lpr* Mice

(A) Anti-dsDNA antibody (n = 19, p = 0.2). (B) Various cytokines and chemokines evaluated by Bio-Plex (n = 6 [MIP1α: n = 8, MCP1 and TNF-α: n = 11], pg/mL). (C) IFNα (n = 6, p = 0.76). (D) HMGB1 (n = 7, p = 0.47). Anti-dsDNA antibody, IFNα, and HMGB1 were evaluated by ELISA. MIP, macrophage inflammatory protein; MCP1, monocyte chemoattractant protein-1.

experiment; however, we were not able to find any beneficial effects for MRL/*lpr* mice (*data not shown*). Although our results could not show absolute efficacy (organomegaly and serological abnormalities did not improve), increased dose and higher blood concentration compared to the previous study might contribute to the efficacy for nephritis.[18] However, HMGB1 was actively translocated from the nucleus in MRL/*lpr* mice, as determined by immunofluorescence; therefore, the dose might be insufficient to antagonize the target in this mouse model. The efficacy of the antagonizing HMGB1 strategy for stroke has been established in animal experiments; however, clinical trials examining such treatments for stroke and autoimmune diseases have yet to be conducted according to ClinicalTrials.gov. Interestingly, there is an HMGB1 antagonist in Chinese herbal medicine, glycyrrhizin, which is approved for clinical practice. The efficacy of glycyrrhizin against various inflammatory conditions has been demonstrated,[44–46] and animal experiments have shown that glycyrrhizin attenuates lupus nephritis.[47] Therefore, glycyrrhizin might mark the first step for clinical application of a treatment strategy that antagonizes HMGB1.

Our anti-HMGB1 mAb failed to improve lymphadenopathy; nevertheless, it demonstrated therapeutic potential against albuminuria in lupus nephritis by inhibiting neutrophil recruitment and NETs without altering autoantibody production. It might be effective especially for membranous lupus nephritis. Although our results demonstrated modest efficacy in MRL/*lpr* mice, it may be dependent on the severity of the disease model in mice and epitope recognition of the antibody. Optimization of dosages and epitope recognition of HMGB1 antibody is required in future translational research in humans.

MATERIALS AND METHODS

Animals

4-week-old female MRL/MpJ-Fas^{lpr}/J (MRL/*lpr*) mice (Jackson Laboratory) were used for animal experiments. Littermates were divided into two groups and then administered with the anti-HMGB1 mAb (5 mg/kg weight; n = 19) or the class-matched control, IgG2a against keyhole limpet hemocyanin (5 mg/kg weight; n = 19), intravenously twice a week from 4 to 15 weeks of age. 24-hr urine samples were collected every 2 weeks from 6 to 16 weeks of age, whereas sera samples were collected every 4 weeks from 8 to 16 weeks of age. Mice were sacrificed at 16 weeks of age and used for the following experiments.

For imaging studies, MRL/*lpr* mice were treated similarly (five mice per group) from 8 to 12 weeks, then sedated and injected with [^{18}F] FAC. PET/CT imaging was performed as previously described, with modifications.[22] In brief, 2-[^{18}F]fluoro-1,3,5-tri-*O*-benzoyl-D-arabinofuranose was prepared by nucleophilic substitution of 2-*O*-(trifluoromethanesulfonyl)-1,3,5-tri-*O*-benzoyl-α-D-ribofuranose, with reactor-produced [^{18}F]fluoride. This ^{18}F-labeled sugar derivative was reacted with a freshly prepared trimethylsilyl-protected derivative of cytosine in dichloroethane. The intermediate analog was then treated with a solution of sodium methoxide in methanol to remove the protecting groups. Following neutralization with

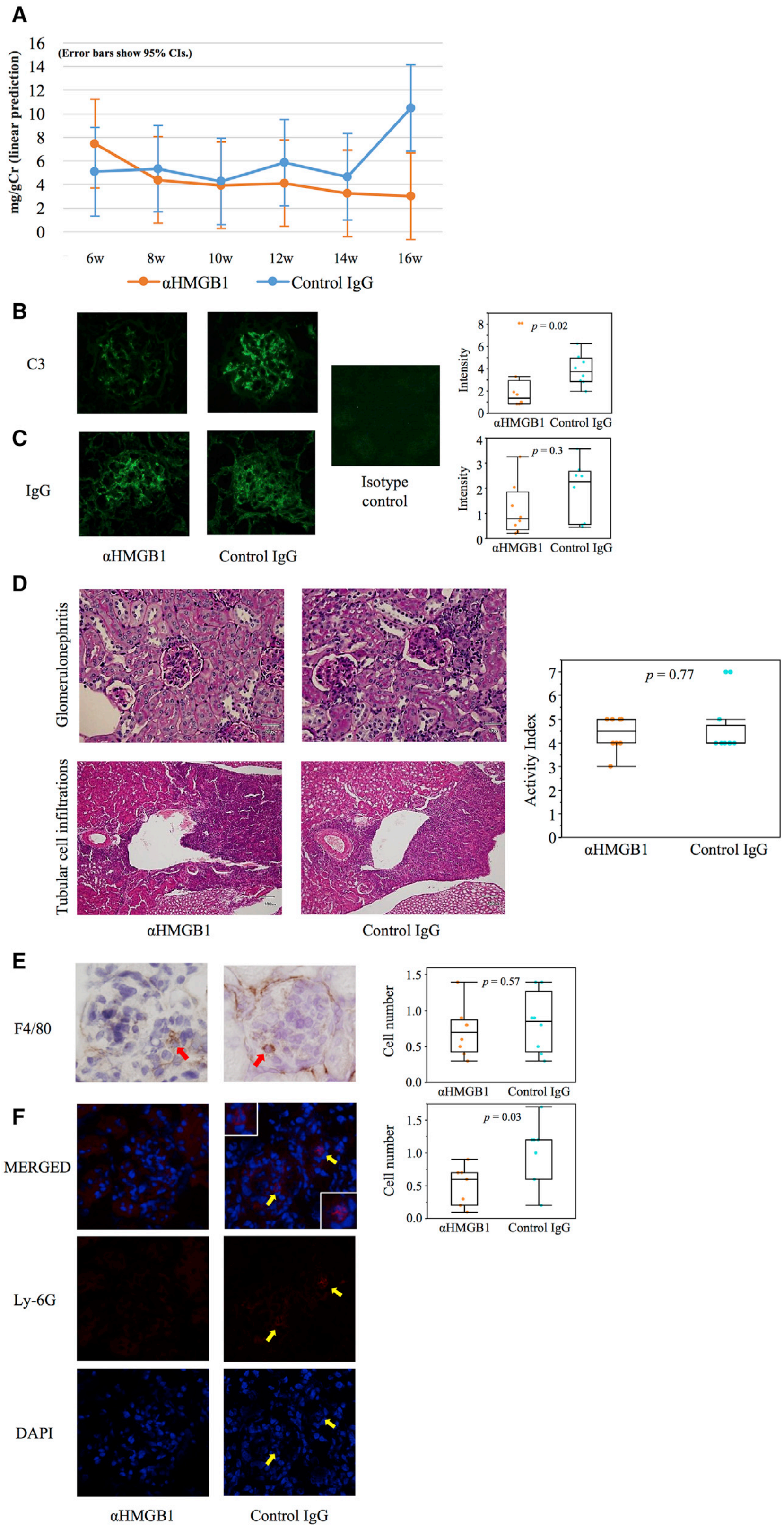

Figure 3. Urinary Albumin Excretion and Renal Pathological Evaluation

(A) Transitions of urine albumin/Cr ratio (model-based adjusted predictions with 95% confidence intervals; n = 19; one value in each group at 6 weeks was missing). At 16 weeks, the 95% confidence intervals of the prediction were not overlapping between the two groups. (B and C) Glomerular depositions of complement (B) (× 200) and IgG (C) (× 200); ten glomeruli were analyzed in each kidney (n = 8). (D) PAS staining of kidney tissues (× 200) and activity indexes; ten glomeruli or tubular regions were analyzed in each kidney (n = 8). (E and F) Glomerular macrophage (E) (× 200, n = 8) and neutrophil infiltration (F) (× 200, n = 7). The number of F4/80-positive or Ly-6G-positive cells was calculated in ten glomeruli per animal, and the mean number of positive cells per glomerulus was used for estimation. Cr, creatinine.

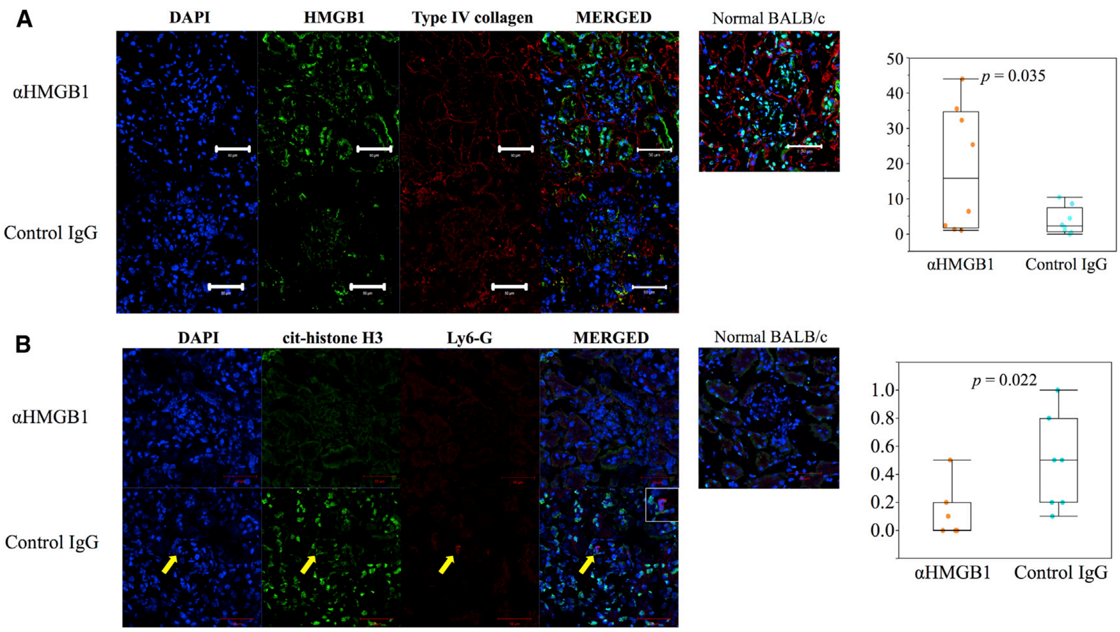

Figure 4. HMGB1 Translocation and NETs

(A) HMGB1 staining of kidneys. HMGB1-positive cells were counted in three to four low-power fields (× 200) per animal, and the mean number was used for estimation (n = 8). (B) NETs in kidneys. The number of NETs was calculated in ten glomeruli per animal, and the mean values per glomerulus were used for estimation (n = 7).

hydrochloric acid, the crude product was purified by semi-preparative high-pressure liquid chromatography, resulting in [^{18}F]FAC with 99% radiochemical purity. PET/CT images were analyzed with PMOD software (PMOD Japan). In addition, the amount of incorporated probe was measured by scintillation counting with the PerkinElmer MicroBeta (PerkinElmer).

These methods were carried out in accordance with the approved guidelines. All experimental protocols were approved by the Animal Care and Use Committee of the Department of Animal Resources, Advanced Science Research Center, Okayama University (OKU-2013112).

Measurement of Urinary Albumin Excretions, Anti-dsDNA Antibodies, HMGB1, Cytokines, and Chemokines

Urinary albumin excretions were measured using the latex agglutination immuno-assay (SHIMA Laboratories) and standardized by urine creatinine concentration. Anti-dsDNA IgG titers, HMGB1 level, and IFNα were measured with mouse anti-dsDNA ELISA kits (Shibayagi), HMGB1 ELISA kits (Shino-Test), and mouse IFNα ELISA kits (Cloud-Clone), respectively. Cytokine and chemokine analyses (except for IFNα) were performed using the cytokine mouse 23-plex Bio-Plex kit (Bio-Rad Laboratories) with a Bio-Plex 200 Luminex machine according to the manufacturer's protocol.

Histopathology and Scoring

Kidney specimens were fixed with 10% buffered formalin and embedded in paraffin. Serial 2-μm sections were stained with H&E or Periodic acid-Schiff (PAS) for histological examination by light microscopy. Activity and chronicity indices were scored according to previously described criteria.[48] Ten renal glomeruli and ten selected tubular areas were examined in each mouse by the CMIC Bioresearch Center.

Immunofluorescence Staining

Kidney tissues (n = 8) were embedded in an optimum cutting temperature compound (Sakura Finetek Japan) and immediately frozen in acetone cooled on dry ice. Frozen sections (4-μm thick) were stained with fluorescein-conjugated anti-mouse IgG (1:50; MP Biomedicals) or anti-mouse C3 (1:50; MP Biomedicals). The brightness of each image file was analyzed using Lumina Vision software (Mitani), as previously described.[49] Briefly, image files were inverted and opened in gray scale mode. IgG or C3 indices were calculated using the following formula: [X (density) × positive area]/glomerular total area, where staining density is indicated by a number from 0 to 256 in gray scale.

For HMGB1 staining, the kidney sections (n = 8) were washed with PBS containing 0.5% Tween 20 (Sigma-Aldrich) and immersed in 10% normal goat serum (Nichirei) containing 1% BSA for 30 min to block nonspecific binding. The sections were incubated overnight with a rabbit anti-HMGB1 Ab (Abcam) in combination with a rat anti-type IV collagen alpha 5 chain mAb (Chondrex) as the primary antibodies at 4°C, and then with Alexa-568-labeled anti-rat IgG (Invitrogen) and Alexa-488-labeled anti-rabbit IgG (Invitrogen) as the secondary antibodies at room temperature (25°C) for 1 hr. Following staining with DAPI, the slides were mounted using fluorescence mounting medium (Dako) and observed under an LSM 780 confocal imaging system (Carl Zeiss). HMGB1-positive cells were counted in

three to four low-power fields (200 × magnification) per animal, and the mean number was used for estimation.

For neutrophil infiltration, the kidney sections (n = 7) were washed with PBS and similarly blocked. The sections were incubated overnight with a rabbit anti-Ly-6G antibody (Clone: 1A8; BioLegend) as the primary antibody at 4°C, and then with Alexa-568-labeled anti-rat IgG as the secondary antibody at room temperature for 1 hr. Nuclear staining and mounting were conducted as described above. The number of Ly-6G-positive cells was calculated in ten glomeruli per animal, and the mean number of positive cells per glomerulus was used for estimation.

The nuclei, Ly-6G, and citrulline R2 + R8 + R17 (cit-histone H3) were stained on 4-μm kidney sections (n = 7) with DAPI and the anti-Ly-6G Ab, followed by Alexa-Fluor-568-labeled anti-goat IgG and anti-cit-histone H3 Ab (Abcam), and followed by Alexa-Fluor-488-labeled anti-rabbit IgG. NETs (both cit-histone-H3- and Ly6G-positive sites) were counted in ten glomeruli per animal, and the mean number of sites per glomerulus was used for the estimation.

Immunoperoxidase Staining

Immunoperoxidase staining was performed as previously described.[50] In brief, macrophage infiltration was analyzed using a monoclonal murine monocyte/macrophage antibody (F4/80; Abcam), followed by an HRP-conjugated goat anti-rat IgG antibody (Merck KGaA). The number of F4/80-stained cells was evaluated similarly to the neutrophil counts.

Real-Time RT-PCR

Total RNA was extracted from kidney tissues using the RNeasy Mini Kit (QIAGEN) according to the manufacturer's instructions and quantitated using a NanoDrop 2000c spectrophotometer (Thermo Scientific). Total RNA was reverse transcribed using the High Capacity cDNA Reverse Transcription Kit (Applied Biosystems). Quantitative real-time PCR was performed using the Step One Plus Real-Time PCR System (Applied Biosystems) with TaqMan Array 96-Well Fast Plates containing specific primers and Universal Master Mix II (Life Technologies) to evaluate the gene expression of IL-1β (Mm00434228_m1), IL-2 (Mm00434256_m1), IL-6 (Mm00446190_m1), IL-10 (Mm00439614_m1), IL-18 (Mm00434225_m1), TNF-α (Mm00443258_m1), IFNα (Mm04207507_gH), ICAM1 (Mm00516023_m1), C-X-C motif chemokine 5 (Mm00436451_g1), HMGB1 (Mm00849805_gH), RAGE (Mm01134790_g1), TLR4 (Mm00445273_m1), TLR7 (Mm00446590_m1), TLR9 (Mm00446193_m1), and NACHT, LRR, and PYD domains containing protein 3 (Mm00840904_m1). The relative mRNA abundance was standardized using GAPDH and 18S mRNA as the invariant control.

Statistical Analysis

Data were compared using the Student's t test or Mann-Whitney *U* test, depending on data distribution. The tests were two-tailed, and the threshold for significance was $p < 0.05$. When analyzing 23-plex Bio-Plex kit or TaqMan Array results, statistical significance was determined at 0.05/23 or 0.05/15 using the Bonferroni correction to adjust for multiple testing. These statistical analyses were performed using the JMP Statistical Package for Windows software, version 9.0.2 (SAS Institute). To evaluate the effects on albuminuria, we estimated the coefficient and 95% confidence intervals (CIs) for local progression using multilevel linear regression analysis (MLA). In MLA, a two-level data structure was employed: level 1 was urine-albumin/creatinine (u-alb/Cr) measurement and level 2 was individual mouse. U-alb/Cr at six time points (6, 8, 10, 12, 14, and 16 weeks) was clustered within the mice. Individual mice were added to the MLA model with a random intercept. We adjusted for the group (anti-HMGB1 versus control) time points (as a dummy variable) and interaction term (group × time points) (Supplemental Information). Model-based adjusted predictions with 95% confidence intervals were compared. MLA was performed using Stata version 14.2 (StataCorp LP).

SUPPLEMENTAL INFORMATION

Supplemental Information includes one figure and one table and can be found with this article online at http://dx.doi.org/10.1016/j.omtm.2017.05.006.

AUTHOR CONTRIBUTIONS

Conceptualization, H.W., K.S.W., and J.W.; Methodology, H.W., K.S.W., K.L., S.H., E.K., S.K., E.M., M.N., and J.W.; Formal Analysis, H.W.; Investigation, H.W., K.S.W., K.L., S.Z., N.T., A.A., F.T., T.H., M.A., T.S., and J.W.; Data Curation, H.W.; Writing – Original Draft, H.W.; Writing – Review and Editing, K.S.W. and J.W.; Visualization, H.W.; Supervision, K.S., E.M., M.N., and J.W.; Project Administration, J.W.; Funding Acquisition, H.W. and K.S.W.

CONFLICTS OF INTEREST

J.W. receives grant support from Astellas, Bayer, Chugai, Daiichi Sankyo, Kissei, Kyowa Hakko Kirin, MSD, Otsuka, Teijin, Torii, Pfizer, Takeda, and Taisho Toyama.

ACKNOWLEDGMENTS

We wish to thank Dr. Toshiharu Mitsuhashi at the Center for Innovative Clinical Medicine of Okayama University Hospital for the multilevel analysis and all technical staff at our department for their assistance. This work was supported by JSPS KAKENHI grant numbers JP16K19600 and JP25860810.

REFERENCES

1. Mok, C.C. (2016). Towards new avenues in the management of lupus glomerulonephritis. Nat. Rev. Rheumatol. *12*, 221–234.
2. Tsokos, G.C. (2011). Systemic lupus erythematosus. N. Engl. J. Med. *365*, 2110–2121.
3. Baumann, I., Kolowos, W., Voll, R.E., Manger, B., Gaipl, U., Neuhuber, W.L., Kirchner, T., Kalden, J.R., and Herrmann, M. (2002). Impaired uptake of apoptotic cells into tingible body macrophages in germinal centers of patients with systemic lupus erythematosus. Arthritis Rheum. *46*, 191–201.
4. Sims, G.P., Rowe, D.C., Rietdijk, S.T., Herbst, R., and Coyle, A.J. (2010). HMGB1 and RAGE in inflammation and cancer. Annu. Rev. Immunol. *28*, 367–388.

5. Harris, H.E., Andersson, U., and Pisetsky, D.S. (2012). HMGB1: a multifunctional alarmin driving autoimmune and inflammatory disease. Nat. Rev. Rheumatol. *8*, 195–202.

6. Scaffidi, P., Misteli, T., and Bianchi, M.E. (2002). Release of chromatin protein HMGB1 by necrotic cells triggers inflammation. Nature *418*, 191–195.

7. Yang, H., Hreggvidsdottir, H.S., Palmblad, K., Wang, H., Ochani, M., Li, J., Lu, B., Chavan, S., Rosas-Ballina, M., Al-Abed, Y., et al. (2010). A critical cysteine is required for HMGB1 binding to Toll-like receptor 4 and activation of macrophage cytokine release. Proc. Natl. Acad. Sci. USA *107*, 11942–11947.

8. Kazama, H., Ricci, J.E., Herndon, J.M., Hoppe, G., Green, D.R., and Ferguson, T.A. (2008). Induction of immunological tolerance by apoptotic cells requires caspase-dependent oxidation of high-mobility group box-1 protein. Immunity *29*, 21–32.

9. Venereau, E., Casalgrandi, M., Schiraldi, M., Antoine, D.J., Cattaneo, A., De Marchis, F., Liu, J., Antonelli, A., Preti, A., Raeli, L., et al. (2012). Mutually exclusive redox forms of HMGB1 promote cell recruitment or proinflammatory cytokine release. J. Exp. Med. *209*, 1519–1528.

10. Tian, J., Avalos, A.M., Mao, S.Y., Chen, B., Senthil, K., Wu, H., Parroche, P., Drabic, S., Golenbock, D., Sirois, C., et al. (2007). Toll-like receptor 9-dependent activation by DNA-containing immune complexes is mediated by HMGB1 and RAGE. Nat. Immunol. *8*, 487–496.

11. Bennett, L., Palucka, A.K., Arce, E., Cantrell, V., Borvak, J., Banchereau, J., and Pascual, V. (2003). Interferon and granulopoiesis signatures in systemic lupus erythematosus blood. J. Exp. Med. *197*, 711–723.

12. Urbonaviciute, V., and Voll, R.E. (2011). High-mobility group box 1 represents a potential marker of disease activity and novel therapeutic target in systemic lupus erythematosus. J. Intern. Med. *270*, 309–318.

13. Abdulahad, D.A., Westra, J., Bijzet, J., Dolff, S., van Dijk, M.C., Limburg, P.C., Kallenberg, C.G., and Bijl, M. (2012). Urine levels of HMGB1 in systemic lupus erythematosus patients with and without renal manifestations. Arthritis Res. Ther. *14*, R184.

14. Zickert, A., Palmblad, K., Sundelin, B., Chavan, S., Tracey, K.J., Bruchfeld, A., and Gunnarsson, I. (2012). Renal expression and serum levels of high mobility group box 1 protein in lupus nephritis. Arthritis Res. Ther. *14*, R36.

15. Taniguchi, N., Kawahara, K., Yone, K., Hashiguchi, T., Yamakuchi, M., Goto, M., Inoue, K., Yamada, S., Ijiri, K., Matsunaga, S., et al. (2003). High mobility group box chromosomal protein 1 plays a role in the pathogenesis of rheumatoid arthritis as a novel cytokine. Arthritis Rheum. *48*, 971–981.

16. Wähämaa, H., Schierbeck, H., Hreggvidsdottir, H.S., Palmblad, K., Aveberger, A.C., Andersson, U., and Harris, H.E. (2011). High mobility group box protein 1 in complex with lipopolysaccharide or IL-1 promotes an increased inflammatory phenotype in synovial fibroblasts. Arthritis Res. Ther. *13*, R136.

17. Zhang, C., Li, C., Jia, S., Yao, P., Yang, Q., and Zhang, Y. (2014). High-mobility group box 1 inhibition alleviates lupus-like disease in BXSB mice. Scand. J. Immunol. *79*, 333–337.

18. Schaper, F., Van Timmeren, M.M., Petersen, A., Horst, G., Bijl, M., Limburg, P.C., Westra, J., and Heeringa, P. (2016). Treatment with anti-HMGB1 monoclonal antibody does not affect lupus nephritis in MRL/lpr mice. Mol. Med. *22*, 12–21.

19. Liu, K., Mori, S., Takahashi, H.K., Tomono, Y., Wake, H., Kanke, T., Sato, Y., Hiraga, N., Adachi, N., Yoshino, T., et al. (2007). Anti-high mobility group box 1 monoclonal antibody ameliorates brain infarction induced by transient ischemia in rats. FASEB J. *21*, 3904–3916.

20. Kanellakis, P., Agrotis, A., Kyaw, T.S., Koulis, C., Ahrens, I., Mori, S., Takahashi, H.K., Liu, K., Peter, K., Nishibori, M., et al. (2011). High-mobility group box protein 1 neutralization reduces development of diet-induced atherosclerosis in apolipoprotein e-deficient mice. Arterioscler. Thromb. Vasc. Biol. *31*, 313–319.

21. Nosaka, N., Yashiro, M., Yamada, M., Fujii, Y., Tsukahara, H., Liu, K., Nishibori, M., Matsukawa, A., and Morishima, T. (2015). Anti-high mobility group box-1 monoclonal antibody treatment provides protection against influenza A virus (H1N1)-induced pneumonia in mice. Crit. Care *19*, 249.

22. Radu, C.G., Shu, C.J., Nair-Gill, E., Shelly, S.M., Barrio, J.R., Satyamurthy, N., Phelps, M.E., and Witte, O.N. (2008). Molecular imaging of lymphoid organs and immune activation by positron emission tomography with a new [18F]-labeled 2′-deoxycytidine analog. Nat. Med. *14*, 783–788.

23. Brinkmann, V., Reichard, U., Goosmann, C., Fauler, B., Uhlemann, Y., Weiss, D.S., Weinrauch, Y., and Zychlinsky, A. (2004). Neutrophil extracellular traps kill bacteria. Science *303*, 1532–1535.

24. Lood, C., Blanco, L.P., Purmalek, M.M., Carmona-Rivera, C., De Ravin, S.S., Smith, C.K., Malech, H.L., Ledbetter, J.A., Elkon, K.B., and Kaplan, M.J. (2016). Neutrophil extracellular traps enriched in oxidized mitochondrial DNA are interferogenic and contribute to lupus-like disease. Nat. Med. *22*, 146–153.

25. Kessenbrock, K., Krumbholz, M., Schönermarck, U., Back, W., Gross, W.L., Werb, Z., Gröne, H.J., Brinkmann, V., and Jenne, D.E. (2009). Netting neutrophils in autoimmune small-vessel vasculitis. Nat. Med. *15*, 623–625.

26. Mantovani, A., Cassatella, M.A., Costantini, C., and Jaillon, S. (2011). Neutrophils in the activation and regulation of innate and adaptive immunity. Nat. Rev. Immunol. *11*, 519–531.

27. Baker, M.J., Pan, D., and Welch, H.C. (2016). Small GTPases and their guanine-nucleotide exchange factors and GTPase-activating proteins in neutrophil recruitment. Curr. Opin. Hematol. *23*, 44–54.

28. McDonald, B., Pittman, K., Menezes, G.B., Hirota, S.A., Slaba, I., Waterhouse, C.C., Beck, P.L., Muruve, D.A., and Kubes, P. (2010). Intravascular danger signals guide neutrophils to sites of sterile inflammation. Science *330*, 362–366.

29. Ferguson, P.J., Lokuta, M.A., El-Shanti, H.I., Muhle, L., Bing, X., and Huttenlocher, A. (2008). Neutrophil dysfunction in a family with a SAPHO syndrome-like phenotype. Arthritis Rheum. *58*, 3264–3269.

30. Denny, M.F., Yalavarthi, S., Zhao, W., Thacker, S.G., Anderson, M., Sandy, A.R., McCune, W.J., and Kaplan, M.J. (2010). A distinct subset of proinflammatory neutrophils isolated from patients with systemic lupus erythematosus induces vascular damage and synthesizes type I IFNs. J. Immunol. *184*, 3284–3297.

31. Zhang, Q., Raoof, M., Chen, Y., Sumi, Y., Sursal, T., Junger, W., Brohi, K., Itagaki, K., and Hauser, C.J. (2010). Circulating mitochondrial DAMPs cause inflammatory responses to injury. Nature *464*, 104–107.

32. Huebener, P., Pradere, J.P., Hernandez, C., Gwak, G.Y., Caviglia, J.M., Mu, X., Loike, J.D., Jenkins, R.E., Antoine, D.J., and Schwabe, R.F. (2015). The HMGB1/RAGE axis triggers neutrophil-mediated injury amplification following necrosis. J. Clin. Invest. *125*, 539–550.

33. Hakkim, A., Fürnrohr, B.G., Amann, K., Laube, B., Abed, U.A., Brinkmann, V., Herrmann, M., Voll, R.E., and Zychlinsky, A. (2010). Impairment of neutrophil extracellular trap degradation is associated with lupus nephritis. Proc. Natl. Acad. Sci. USA *107*, 9813–9818.

34. Lindau, D., Mussard, J., Rabsteyn, A., Ribon, M., Kötter, I., Igney, A., Adema, G.J., Boissier, M.C., Rammensee, H.G., and Decker, P. (2014). TLR9 independent interferon α production by neutrophils on NETosis in response to circulating chromatin, a key lupus autoantigen. Ann. Rheum. Dis. *73*, 2199–2207.

35. Li, P., Li, M., Lindberg, M.R., Kennett, M.J., Xiong, N., and Wang, Y. (2010). PAD4 is essential for antibacterial innate immunity mediated by neutrophil extracellular traps. J. Exp. Med. *207*, 1853–1862.

36. Knight, J.S., Zhao, W., Luo, W., Subramanian, V., O'Dell, A.A., Yalavarthi, S., Hodgin, J.B., Eitzman, D.T., Thompson, P.R., and Kaplan, M.J. (2013). Peptidylarginine deiminase inhibition is immunomodulatory and vasculoprotective in murine lupus. J. Clin. Invest. *123*, 2981–2993.

37. Knight, J.S., Subramanian, V., O'Dell, A.A., Yalavarthi, S., Zhao, W., Smith, C.K., Hodgin, J.B., Thompson, P.R., and Kaplan, M.J. (2015). Peptidylarginine deiminase inhibition disrupts NET formation and protects against kidney, skin and vascular disease in lupus-prone MRL/lpr mice. Ann. Rheum. Dis. *74*, 2199–2206.

38. Tadie, J.M., Bae, H.B., Jiang, S., Park, D.W., Bell, C.P., Yang, H., Pittet, J.F., Tracey, K., Thannickal, V.J., Abraham, E., et al. (2013). HMGB1 promotes neutrophil extracellular trap formation through interactions with Toll-like receptor 4. Am. J. Physiol. Lung Cell. Mol. Physiol. *304*, L342–L349.

39. Huang, H., Tohme, S., Al-Khafaji, A.B., Tai, S., Loughran, P., Chen, L., Wang, S., Kim, J., Billiar, T., Wang, Y., et al. (2015). Damage-associated molecular pattern-activated neutrophil extracellular trap exacerbates sterile inflammatory liver injury. Hepatology *62*, 600–614.

40. Bruchfeld, A., Wendt, M., Bratt, J., Qureshi, A.R., Chavan, S., Tracey, K.J., Palmblad, K., and Gunnarsson, I. (2011). High-mobility group box-1 protein (HMGB1) is

increased in antineutrophilic cytoplasmatic antibody (ANCA)-associated vasculitis with renal manifestations. Mol. Med. *17*, 29–35.

41. Ma, Y.H., Ma, T.T., Wang, C., Wang, H., Chang, D.Y., Chen, M., and Zhao, M.H. (2016). High-mobility group box 1 potentiates antineutrophil cytoplasmic antibody-inducing neutrophil extracellular traps formation. Arthritis Res. Ther. *18*, 2.
42. Kokkola, R., Li, J., Sundberg, E., Aveberger, A.C., Palmblad, K., Yang, H., Tracey, K.J., Andersson, U., and Harris, H.E. (2003). Successful treatment of collagen-induced arthritis in mice and rats by targeting extracellular high mobility group box chromosomal protein 1 activity. Arthritis Rheum. *48*, 2052–2058.
43. Ostberg, T., Kawane, K., Nagata, S., Yang, H., Chavan, S., Klevenvall, L., Bianchi, M.E., Harris, H.E., Andersson, U., and Palmblad, K. (2010). Protective targeting of high mobility group box chromosomal protein 1 in a spontaneous arthritis model. Arthritis Rheum. *62*, 2963–2972.
44. Xiong, X., Gu, L., Wang, Y., Luo, Y., Zhang, H., Lee, J., Krams, S., Zhu, S., and Zhao, H. (2016). Glycyrrhizin protects against focal cerebral ischemia via inhibition of T cell activity and HMGB1-mediated mechanisms. J. Neuroinflammation *13*, 241.
45. Bangert, A., Andrassy, M., Müller, A.M., Bockstahler, M., Fischer, A., Volz, C.H., Leib, C., Göser, S., Korkmaz-Icöz, S., Zittrich, S., et al. (2016). Critical role of RAGE and HMGB1 in inflammatory heart disease. Proc. Natl. Acad. Sci. USA *113*, E155–E164.
46. Tian, S., Zhang, L., Tang, J., Guo, X., Dong, K., and Chen, S.Y. (2015). HMGB1 exacerbates renal tubulointerstitial fibrosis through facilitating M1 macrophage phenotype at the early stage of obstructive injury. Am. J. Physiol. Renal Physiol. *308*, F69–F75.
47. Li, X., Yue, Y., Zhu, Y., and Xiong, S. (2015). Extracellular, but not intracellular HMGB1, facilitates self-DNA induced macrophage activation via promoting DNA accumulation in endosomes and contributes to the pathogenesis of lupus nephritis. Mol. Immunol. *65*, 177–188.
48. Austin, H.A., 3rd, Muenz, L.R., Joyce, K.M., Antonovych, T.T., and Balow, J.E. (1984). Diffuse proliferative lupus nephritis: identification of specific pathologic features affecting renal outcome. Kidney Int. *25*, 689–695.
49. Nasu, T., Maeshima, Y., Kinomura, M., Hirokoshi-Kawahara, K., Tanabe, K., Sugiyama, H., Sonoda, H., Sato, Y., and Makino, H. (2009). Vasohibin-1, a negative feedback regulator of angiogenesis, ameliorates renal alterations in a mouse model of diabetic nephropathy. Diabetes *58*, 2365–2375.
50. Terami, N., Ogawa, D., Tachibana, H., Hatanaka, T., Wada, J., Nakatsuka, A., Eguchi, J., Horiguchi, C.S., Nishii, N., Yamada, H., et al. (2014). Long-term treatment with the sodium glucose cotransporter 2 inhibitor, dapagliflozin, ameliorates glucose homeostasis and diabetic nephropathy in db/db mice. PLoS ONE *9*, e100777.

OMTM, Volume *6*

Supplemental Information

Anti-high Mobility Group Box 1 Antibody Ameliorates Albuminuria in MRL/*lpr* Lupus-Prone Mice

Haruki Watanabe, Katsue S. Watanabe, Keyue Liu, Sumie Hiramatsu, Sonia Zeggar, Eri Katsuyama, Noriko Tatebe, Akiya Akahoshi, Fumiaki Takenaka, Takahisa Hanada, Masaru Akehi, Takanori Sasaki, Ken-ei Sada, Eiji Matsuura, Masahiro Nishibori, and Jun Wada

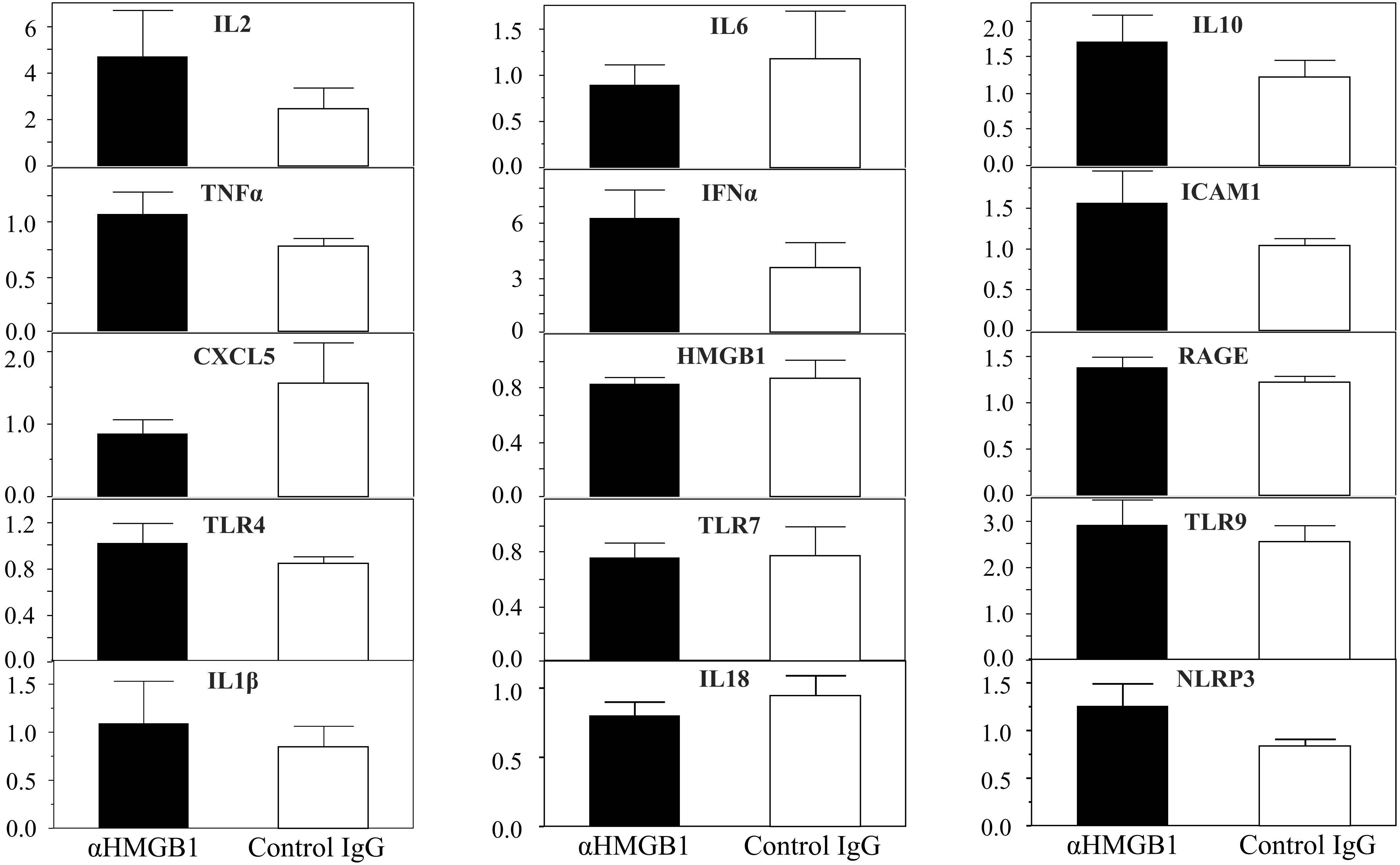
IL2
IL6
IL10
TNFα
IFNα
ICAM1
CXCL5
HMGB1
RAGE
TLR4
TLR7
TLR9
IL1β
IL18
NLRP3
αHMGB1
Control IgG
αHMGB1
Control IgG
αHMGB1
Control IgG

Supplemental Figure 1. Renal mRNA expression

Relative mRNA expression was evaluated by real-time RCR (n = 9 [IL2: n = 6, IL10, TNF α , ICAM, TLR4: n = 10]). Relative mRNA abundance was standardized using GAPDH and 18S mRNA as the invariant control. IL, interleukin; TNF, tumor necrosis factor; IFN, interferon; ICAM1, intercellular adhesion molecule 1; CXCL5, C-X-C motif chemokine 5; HMGB1, high mobility group box 1; RAGE, receptor for advanced glycation endproducts; TLR, toll-like receptor; NLRP3, NACHT, LRR and PYD domains-containing protein 3.
